# First Report on Abortion Caused by *Salmonella enterica* subsp. *enterica* Serovar Enteritidis in Water Buffalo (*Bubalus bubalis*)

**DOI:** 10.3389/fvets.2022.894154

**Published:** 2022-07-11

**Authors:** Luisa D'Angelo, Rubina Paradiso, Domenico Alfano, Marita Georgia Riccardi, Giorgia Borriello, Giorgio Galiero

**Affiliations:** ^1^Istituto Zooprofilattico Sperimentale del Mezzogiorno, National Reference Centre for Hygiene and Technologies of Water Buffalo Farming and Productions, Salerno, Italy; ^2^Department of Animal Health, Istituto Zooprofilattico Sperimentale del Mezzogiorno, Unit of Genetics Genomics and Bioinformatics, Naples, Italy

**Keywords:** water buffalo (*Bubalus bubalis*), *Salmonella* Enteritidis, abortion, whole genome sequencing, microbial genomics

## Abstract

*Salmonella enterica* subsp. *enterica* Serovar Enteritidis is one of the major pathogens associated with enteric diseases in animals and humans. Thus, due to the importance of *Salmonella* spp. infections for animal production and public health, the aim of the present study was to describe the first detection of *S. enteritidis* in an aborted water buffalo fetus in southern Italy by characterizing the phylogroup profile and the antimicrobial susceptibility of the isolated pathogenic strains. The different clinical manifestations of salmonellosis in animals include diarrhea, abortion, pneumonia, septic arthritis, meningitis, and others, depending on the virulence of the serovars, infectious dose, and host immunity. This study reports the first case of abortion caused by *Salmonella enterica* subsp *enterica* serovar Enteritidis in water buffalo (*Bubalus bubalis*) in the Campania region, southern Italy. Complete necropsy was performed on the aborted water buffalo fetus under study, and samples and swabs from different organs were collected. Samples were processed by microbiological and molecular analyses to detect bacterial, viral, and protozoarian pathogens possibly responsible for abortion. Whole genome sequencing (WGS) was carried out to further characterize the isolated *S*. Enteritidis strain. Our findings highlight the crucial role of *S*. Enteritidis as a potential abortive agent in water buffalo and its presence should therefore be investigated in cases of bubaline abortion.

## Introduction

*Salmonella enterica* is one of the most common pathogens responsible for human and animal bacterial intestinal infections. The main subspecies of this species is *enterica*, which includes more than 2,600 serovars (1). *Salmonella enterica* subsp. *enterica* serovars exhibit broadly diverse phenotypes characterized by different infectious patterns, reservoirs, vectors, and host spectrum (2). Despite the high number of *S. enterica* serovars, only one or a few are associated with the majority of cases of illness in mammalian species, with Typhimurium and Dublin predominating in cattle (3–5). These serotypes show different degrees of host adaptation, with *S*. Typhimurium most frequently associated with gastrointestinal disease in young calves (younger than 8 weeks), and S. Dublin inducing diseases both in young and adult animals (3–5). The severity of the disease is determined by host-pathogen interactions, highly influenced by several factors including the route of infection, the infectious dose, the virulence of the pathogen, natural or acquired host resistance factors, and the possible presence of other pathogens (6, 7).

In water buffalo calves (*Bubalus bubalis*), *Salmonella* induces severe gastrointestinal lesions, diarrhea, hyperthermia, and severe dehydration (6, 8). The major source of infection in water buffalo herds is represented by carrier animals, shedding heavy loads of *Salmonella* through feces (9–11). Other possible sources of infection include contaminated forages and water, as well as rodents, wild winged animals, insects and man (12). The S. *enterica* serovars most frequently isolated from water buffalo are Typhimurium, Muenster and Give (13). Moreover, this pathogen can harbor a variety of virulence factors, with different locations in the genome, such as *Salmonella* pathogenicity islands (SPIs), prophage regions, or virulence plasmids (7). The complex pattern of virulence factors can increase the ability of *Salmonella* to cause systemic infection and consequently to induce abortion in many animal species. Indeed *Salmonella enterica* can infect the fetus by passing through the placental barrier, thus causing abortion, stillbirth, and even pre-term and neonatal infections in animals (14). Abortion due to *Salmonella* spp. has already been described in cattle and small ruminants (15–17), however it has never been recorded in water buffalo. Therefore, the aim of this study was to describe for the first time a case of abortion in water buffalo caused by *S. enterica* subsp. *enterica* serovar Enteritidis.

## Methods

### Herd of Origin

The herd where the abortion occurred consists of about 600 animals, grazing outdoor in the corrals with an off-paddock system. It regularly carries out deratting interventions and is fully fenced. The water supply is represented by well water, regularly subjected to microbiological analysis to verify its potability. In the years preceding the abortion, a vaccination protocol was carried out on adult animals exclusively for Rotavirus, Coronavirus and *E. coli* K 99. During the last 2 years only sporadic abortions occurred in the farm, none of which related to *Salmonella* spp., except that reported in the present study. Moreover, in the same period, mortality episodes recorded among calves could not be attributed to *Salmonella* outbreaks, rather to wrong management procedures. The herd is often flied over by many wild birds.

### Anatomopathological and Microbiological Examinations

The study was carried out on an aborted fetus delivered by a water buffalo heifer bred in a herd located in the Campania region, southern Italy. The abortion occurred at the fifth month of gestation. Complete necropsy was performed within 24 h after death by veterinarians from the Istituto Zooprofilattico Sperimentale del Mezzogiorno (IZSM), Salerno, Italy. When the abdominal cavity was opened the subject exhibited severe abdominal and moderate pleural serohematic effusions, with the presence of mild pericardial serohematic fuid. Samples and swabs from the celomatic organs were collected according to standard protocols (18). Samples of abomasum, lungs, liver, spleen, heart, and kidneys were collected and investigated for the presence of the pathogens most frequently associated with abortion in water buffalo, including Bovine herpesvirus 1 (BHV-1), Bovine viral diarrhea virus (BVDV), *Brucella* spp., *Campylobacter fetus, Listeria* spp., *Salmonella* spp., Bovine herpesvirus 4 (BHV-4), *Chlamydophila abortus, Coxiella burnetii, Neospora caninum, Toxoplasma gondii*, and *Leptospira* spp. (19). Feces sampled from the heifer were tested for the presence of *Salmonella* spp. by microbiological method (20). Table 1 reports the investigated abortive agents for each tissue sample by cultural and/or molecular methods (20, 21). Due to its small dimensions, spleen was homogenized and entirely used for the bacteriological research only of *Brucella* spp., while the presence of *Salmonella* was investigated on the liver.

**Table 1:** Investigated abortive agents for each tested organ.

**Matrix**	**Pathogen**	**Test**
Lungs	Bovine herpesvirus 1 (BHV-1), Bovine herpesvirus 4 (BHV-4), *Listeria* spp., *Chlamydophila abortus, Coxiella burnetii*	PCR
Liver	Bovine herpesvirus 1 (BHV-1), *Campylobacter fetus, Listeria* spp., *Chlamydophila abortus, Coxiella burnetii*	
Spleen	Bovine viral diarrhea virus (BVDV), Bovine herpesvirus 4 (BHV-4)	
Abomasum content	*Campylobacter fetus, Listeria* spp., *Chlamydophila abortus, Coxiella burnetii*	
Heart	*Neospora caninum, Toxoplasma gondii*	
Kidney	*Leptospira* spp.	
Lungs	*Brucella* spp., *Listeria* spp.	Bacteriological
Liver	*Salmonella* spp.	
Spleen	*Brucella* spp.	
Abomasum content	*Brucella* spp., *Campylobacter fetus, Listeria* spp.	

### Microbial Identification

*Salmonella enterica* isolation and identification were performed according to UNI EN ISO 6579-1:2020 (22). The isolated *S*. Enteritidis was serotyped according to the Kaufmann-White scheme (23). Antimicrobial susceptibility testing (AST) was performed using the Kirby-Bauer disk diffusion method. The antibiotics tested were nalidixic acid, ampicillin, cefotaxime, ciprofloxacin, chloramphenicol, gentamicin, kanamycin, streptomycin, sulphonamide, tetracycline, sulfamethoxazole/trimethoprim, colistin, amoxicillin-clavulanic acid, enrofloxacin, cephalothin, and ceftazidime.

### WGS-Based Characterization

The isolated *S*. Enteritidis was processed for genomic DNA isolation by using the QIAamp DNA mini kit (Qiagen, Hilden, Germany) following manufacturer's instructions. DNA concentration was evaluated using high-sensitivity Qubit™ fluorometer (ThermoFisher Scientific, Waltham, MA, USA). Approximately 150 ng of genomic DNA were used for whole genome sequencing. A 400 bp fragment DNA library was prepared using the Ion Xpress Fragment Library kit (Life Technologies, Carlsbad, CA, USA) following manufacturer's instructions. Sequencing reaction was carried out on an Ion Gene Studio S5 platform (ThermoFisher Scientific, Waltham, MA, USA). A coverage of approximately 30X was calculated. The raw sequencing reads were analyzed using FastQC, and low-quality sequences were removed by using PRINSEQ lite. High-quality sequences were assembled using SPAdes (24) version 3.15.0. Sequences assembly produced 46 contigs for a total length of 4,682,282 bp, with a mean contig length of 102,386 nucleotides (maximum contig length: 454,631 nucleotides, and minimum contig length: 537 nucleotides) and an L50 contig value of 9, with an N50 length value of 197,095. Contigs were aligned with the reference sequence NZ_CP075122.1 using ABACAS version 1.3.1. The obtained sequence of the bacterial genome was deposited in GenBank with accession number CP088146.1 and was analyzed with the bioinformatic tools available on the Center for Genomic Epidemiology (CGE) website https://www.genomicepidemiology.org.

## Results

Cultural and biochemical investigations identified the pathogen isolated from the analyzed fetus as *Salmonella enterica* subsp. *enterica* serovar Enteritidis *9,12:g.m*:-. The other investigated bacterial, viral and protozoarian pathogens were not detected. No other possible cause of abortion was identified. Feces sampled from the heifer resulted negative for the presence of *Salmonella* spp. when processed by microbiological method. The isolated strain exhibited resistance to the class of aminoglycosides when challenged with the Kirby-Bauer disk diffusion test.

Whole Genome Sequencing of the isolated *S*. Enteritidis strain allowed to perform a molecular characterization of the pathogen under study. Specifically, this strain exhibited the MLST profile type 11 and harbored two plasmids, IncFIB(S) and IncFII(S). Moreover, the molecular characterization confirmed the antigenic profile obtained by the Kaufmann-White scheme and resistance to aminoglycoside identified by microbiological methods. The strain under study displayed the presence of 148 genes coding for virulence factors (Supplementary Table 1). Most of these virulence genes were involved in mechanisms responsible for fimbrial and non-fimbrial adherence determinants and secretion systems. The characterized strain also exhibited the presence of several *Salmonella* Pathogenicity Islands (SPIs), including SPI-1, SPI-2, SPI-3, SPI-5, SPI-9, SPI-10, SPI-12, SPI-13, and SPI-14. The pathogen finder tool indicated that the analyzed strain was predicted to be a possible human pathogen with a probability of 94% based on the presence of the identified virulence genes.

## Discussion

This report describes for the first time a case of abortion associated with *S*. Enteritidis in water buffalo. Abortion is a major concern in water buffalo dairy farms, with potentially significant economic losses. Causes of abortion for this animal species, as well as for bovine, can be either infectious or non-infectious. Among the infectious causes of abortion, in addition to frequently described pathogens, such as *Brucella* spp., Bovine Viral Diarrhea Virus, Bovine Herpesvirus 1, *N. caninum, Toxoplasma gondii, Campylobacter fetus, C. burnetii, Chlamydia* spp. and *Leptospira*, other less common pathogens, such as some *Salmonella* serovars can also be included (16, 17, 25). In particular, in cattle, *S*. Dublin has been described as a pathogen responsible for abortion (17, 25, 26).

In water buffalo herds *Salmonella* Enteritidis is a matter of concern since it can cause serious economic losses in livestock and is a zoonotic agent responsible for foodborne illness (8, 27). Indeed, the pathogens either causing reproductive disorders and infectious diseases in water buffaloes and/or transmitted through their products pose a serious risk to the overall livestock production because they affect animal health status and milk production. In water buffalo, *Salmonella* is a major pathogen because it induces calf diarrhea leading to early-age calf-mortality (28) and can be transmitted through animal products thus contaminating the food chain. *Salmonella* can enter and survive in the farm environment for long periods of time. The presence of this pathogen is widespread in water buffalo herds globally (13, 29–32) and its prevalence can be up to 26% in farm environment, as reported by a recent study (32). In farm animals information on the circulating pathogens and, in case of abortion, the correct identification of the etiological cause, could lessen the impact of the related pathologies.

The *S*. Enteritidis strain under study exhibited a high number of genes coding for virulence factors. Consistently with the abortion as the outcome of the infection, this strain harbored several virulence factors known to be responsible for the ability of some *Salmonella* strains to escape the host immune system, overcome the intestinal wall and gain systemic circulation, such as Type I fimbriae and genes located on diverse SPIs (33, 34). This strain was instead lacking some other virulence factors also known to be implicated in the systemic infection of *Salmonella*, such as those associated with the capsule. This evidence suggests that the pathogenesis of this microorganism is complex and likely modulated by a high number of genes, many of which have not yet been fully elucidated in regard to their role in the course of the infection. Moreover, the host-pathogen interaction also plays a fundamental role in the outcome of the infection, with particular regard to the animal species considered in this study. Our data suggest that in water buffalo *S*. Enteritidis might exhibit a particular tropism for the reproductive apparatus based on the evidence that the heifer under study showed the absence of gastroenteric disease either before or after abortion, as well as the absence of the bacterium in her feces. Finally, although *Salmonella* spp. is frequently characterized by multi-antibiotic resistance (35), the strain under study exhibited only resistance to aminoglycoside, while was susceptible to all the other tested antibiotics. In general, the analysis of bacteria from food animals, foods, and clinically ill humans with the subsequent evaluation of antimicrobial resistance trends throughout the food production and supply chain is a primary requirement for an integrated and efficient surveillance program.

For the first time we have shown that *S*. Enteritidis causes abortion in water buffalo. This confirms the health risk posed by *Salmonella enterica* to farm animals and represents a further loss for the water buffalo marketing sector. In light of these findings, considering the zoonotic potential of *Salmonella enterica* and its ability to act as a potential abortive agent in water buffalo, its presence should always be investigated in cases of bubaline abortion. More research is needed to understand why few *Salmonella* serovars are responsible for the majority of animal diseases and demonstrate such unique reservoirs and pathogenesis. With a better understanding of serovar specificity and pathogenicity, effective strategies could be set up to control *Salmonella* in livestock farms.

## Data Availability Statement

The datasets presented in this study can be found in online repositories. The names of the repository/repositories and accession number(s) can be found in the article/Supplementary Material.

## Ethics Statement

Ethical review and approval was not required for the animal study because the experimental Zooprophylactic institutes (IIZZSSMM) are official laboratories designed by the Italian Ministry of Health that are involved in epidemiological surveillance, animal health, food, and feed safety and diagnostics. For this reason, ethics approval was deemed unnecessary in agreement with institutional policy and national regulations. Written informed consent for participation was not obtained from the owners because the study was conducted during institutional activities.

## Author Contributions

LD'A, RP, DA, MR, GB, and GG drafted the manuscript. LD, GB, and GG conceded and revised the study. DA, RP, and MR conducted the microbiological and molecular analyses. All authors contributed to the article and approved the submitted version.

## Conflict of Interest

The authors declare that the research was conducted in the absence of any commercial or financial relationships that could be construed as a potential conflict of interest.

## Publisher's Note

All claims expressed in this article are solely those of the authors and do not necessarily represent those of their affiliated organizations, or those of the publisher, the editors and the reviewers. Any product that may be evaluated in this article, or claim that may be made by its manufacturer, is not guaranteed or endorsed by the publisher.

## References

[1] Ryan MP O'Dwyer J Adley CC. (2017). Evaluation of the complex nomenclature of the clinically and veterinary significant pathogen *Salmonella*. Biomed Res Int. 3782182. 10.1155/2017/3782182 28540296 PMC5429938

[2] Vila Nova M Durimel K La K Felten A Bessières P Mistou MY. Genetic and metabolic signatures of *Salmonella enterica* subsp. *enterica* associated with animal sources at the pangenomic scale. BMC Genomics. (2019) 20:814. 10.1186/s12864-019-6188-x 31694533 PMC6836353

[3] Bäumler AJ Tsolis RM Ficht TA Adams LG. Evolution of host adaptation in *Salmonella enterica*. Infect Immun. (1998). 66:4579–87. 10.1128/IAI.66.10.4579-4587.1998 9746553 PMC108564

[4] Rice DH Besser TE Hancock DD. Epidemiology and virulence assessment of *Salmonella dublin*. Vet Microbiol. (1997) 56:111–24. 10.1016/S0378-1135(96)01352-1 9228687

[5] Roy JHB. Salmonellosis. In: Roy JHB, editor. The Calf. 1 Management of Health. London: Butterworths (1990). p. 118–30.

[6] Adlakha SC Sharma SN. Infectious diseases. In: Tulloh NM, Holmes JHG, editor. World Animal Science C6, Buffalo Productions. Parkville: Elsevier (1992) p. 271–303.

[7] Barrow PA Jones MA Thomson N. Salmonella. In: Gyles CL, Prescott JF, Songer JG, Thoen CO, editor. Pathogenesis of Bacterial Infections in Animals. Ames: Blackwell Publishing (2010) p. 231–265. 10.1002/9780470958209.ch14

[8] Fagiolo A Roncoroni C Lai O Borghese A. Buffalo pathologies. In: Borghese A, editor. Buffalo Production Research. Rome: FAO Regional Office for Europe Inter-Regional Cooperative Research Network on Buffalo (2005).

[9] Gosling RJ Mueller-Doblies D Martelli F Nunez-Garcia J Kell N Rabie A. Observations on the distribution and persistence of monophasic *Salmonella* Typhimurium on infected pig and cattle farms. Vet Microbiol. (2018) 227:90–6. 10.1016/j.vetmic.2018.10.032 30473358

[10] Edrington TS Garcia Buitrago JA Hagevoort GR Loneragan GH Bricta-Harhay DM Callaway TR. Effect of waste milk pasteurization on fecal shedding of *Salmonella* in preweaned calves. J Dairy Sci. (2018) 101:9266–74. 10.3168/jds.2018-14668 30077443

[11] Pereira R Williams DR Rossitto P Adaska J Okello E Champagne J. Association between herd management practices and antimicrobial resistance in *Salmonella* spp. from cull dairy cattle in Central California. Peer J. (2019) 7:e6546. 10.7717/peerj.6546 30923650 PMC6431540

[12] Quinn PJ Markey B Leonard FC FitzPatrick ES Fanning S Hartigan PJ. Veterinary Microbiology and Microbial Disease. 2nd Edn. West Sussex: Wiley Blackwell (2011).

[13] Borriello G Lucibelli MG Pesciaroli M Carullo MR Graziani C Ammendola S. Diversity of *Salmonella* spp. serovars isolated from the intestines of water buffalo calves with gastroenteritis. BMC Vet Res. (2012) 8:201. 10.1186/1746-6148-8-201 23098237 PMC3514206

[14] Anderson ML. Infectious causes of bovine abortion during mid- to late-gestation. Theriogenology. (2007) 68:474–86. 10.1016/j.theriogenology.2007.04.001 17467787

[15] Strugnell BW Bennett G Davies RH Horton RA. Bovine abortion associated with *Salmonella* 9,12:-:-NM in a UK dairy herd. Vet Rec. (2011) 169:208. 10.1136/vr.d4301 21778145

[16] Derdour SY Hafsi F Azzag N Tennah S Laamari A China B. Prevalence of the main infectious causes of abortion in dairy cattle in Algeria. J Vet Res. (2017) 61:337–43. 10.1515/jvetres-2017-0044 29978092 PMC5894425

[17] Barkallah M Gharbi Y Hassena AB Slima AB Mallek Z Gautier M. Survey of infectious etiologies of bovine abortion during mid- to late gestation in dairy herds. PLoS ONE. (2014) 9:e91549. 10.1371/journal.pone.0091549 24662769 PMC3963856

[18] Severidt JA Madden DJ Mason GL Garry FB Gould DH. Dairy Cattle Necropsy Manual. Fort Collins, CO in Integrated Livestock Management. Colorado State University (2002). Available online at: http://www.cvmbs.colostate.edu/ilm/proinfo/necropsy/notes/index.html (accessed September 15, 2021)

[19] Esposito C Cardillo L Borriello G Ascione G Valvini O Galiero G. First detection of *Listeria monocytogenes* in a buffalo aborted foetus in Campania Region (Southern Italy). Front Vet Sci. (2021) 7:571654. 10.3389/fvets.2020.571654 33644140 PMC7902923

[20] OIE-World Organization for Animal Health. Manual of Diagnostic Tests and Vaccines for Terrestrial Animals. 8th Edn. (2018). Available online at: https://www.oie.int/fileadmin/Home/eng/Health_standards/tahm/3.04.11_IBR_IPV.pdf (accessed November 14, 2019).

[21] Tramuta C Lacerenza D Zoppi S Goria M Dondo A Ferroglio E. Development of a set of multiplex standard polymerase chain reaction assays for the identification of infectious agents from aborted bovine clinical samples. J Vet Diagn Invest. (2011) 23:657–64. 10.1177/1040638711407880 21908306

[22] UNI EN ISO 6579-1:2017/Amd 1:2020 Microbiology of the food chain — Horizontal method for the detection enumeration enumeration and serotyping of Salmonella — Part 1: Detection of Salmonella spp. — Amendment 1: Broader range of incubation temperatures, amendment to the status of Annex D, and correction of the composition of MSRV and SC.

[23] Grimont PAD Weill FX. Antigenic Formulae of the Salmonella serovars. 9th Edn. Paris: WHO Collaborating Centre for Reference and Research on Salmonella (2007).

[24] Bankevich A Nurk S Antipov D Gurevich AA Dvorkin M Kulikov AS. SPAdes: a new genome assembly algorithm its applications to single-cell sequencing. J. Comput. Biol. (2012) 19:455–77. 10.1089/cmb.2012.0021 22506599 PMC3342519

[25] Sodoma E Mitterhuemer S Krassnig G Stellnberger K Reisp K Schmoll F. [Infectious causes of abortions in cattle - own experiences and investigations from 2018 (January-September)]. Tierarztl Prax Ausg G Grosstiere Nutztiere. (2019) 47:143–50. 10.1055/a-0896-0945 31212340

[26] Carrique-Mas JJ Willmington JA Papadopoulou C Watson EN Davies RH. Salmonella infection in cattle in Great Britain, 2003 to 2008. Vet Rec. (2010) 167:560–5. 10.1136/vr.c4943 21257417

[27] Abulreesh HH. Salmonellae in the environment. In: Annous B, Gurtler JB, editors. Salmonella—Distribution, Adaptation, Control Measures and Molecular Technologies. InTech (2012). p. 19–50.

[28] Amrousi SEL Nafie EK Rehewi MEL Mottilib AA. Studies on enteritis in buffalo calves in Assiut. J Egypt Vet Med Assoc. (1971) 31:219–25.

[29] Santana AM da Silva DG Maluta RP Pizauro LJL Simplício KMMG Santana CH. Comparative analysis using pulsed-field gel electrophoresis highlights a potential transmission of *Salmonella* between asymptomatic buffaloes and pigs in a single farm. Front Vet Sci. (2020) 7:552413. 10.3389/fvets.2020.552413 33240945 PMC7683720

[30] Ahmed LM Sayed ASM ElKader HAA Faddan NHA Al Hosary AAT. Phylogenetic analysis of *Salmonella* species isolated from cows, buffaloes, and humans based on gyrB gene sequences. Trop Anim Health Prod. (2020) 52:1487–92. 10.1007/s11250-019-02155-y 31898024

[31] Bantawa K Sah SN Subba Limbu D Subba P Ghimire A. Antibiotic resistance patterns of *Staphylococcus aureus, Escherichia coli, Salmonella, Shigella* and *Vibrio* isolated from chicken, pork, buffalo and goat meat in eastern Nepal. BMC Res Notes. (2019) 12:766. 10.1186/s13104-019-4798-7 31752992 PMC6873459

[32] Rodriguez A Pangloli P Richards HA Mount JR Draughon FA. Prevalence of *Salmonella* in diverse environmental farm samples. J Food Prot. (2006) 69:2576–80. 10.4315/0362-028X-69.11.2576 17133798

[33] Dos Santos AMP Ferrari RG Conte-Junior CA. Virulence factors in *Salmonella* typhimurium: the sagacity of a bacterium. Curr Microbiol. (2019) 76:762–73. 10.1007/s00284-018-1510-4 29785632

[34] Bao H Wang S Zhao JH Liu SL. *Salmonella* secretion systems: differential roles in pathogen-host interactions. Microbiol Res. (2020) 241:126591. 10.1016/j.micres.2020.126591 32932132

[35] McDermott PF Zhao S Tate H. Antimicrobial resistance in nontyphoidal *Salmonella*. Microbiol Spectr. (2018) 6:1–26. 10.1128/microbiolspec.ARBA-0014-2017 30027887 PMC11633595

